# AI-Enhanced Surgical Decision-Making in Orthopedics: From Preoperative Planning to Intraoperative Guidance and Real-Time Adaptation

**DOI:** 10.7759/cureus.92762

**Published:** 2025-09-19

**Authors:** Ahmed Elkohail, Ali Soffar, Ahmed M Khalifa, Ibrahim Omar, Maryam Mosaad, Mostafa Abdulaziz, Ahmed Elsaket, Hafsa S Panhwer, Momen Abdelglil, Mahmoud Teama, Ahmed Swealem

**Affiliations:** 1 Orthopaedics and Trauma, Princess Royal University Hospital, King's College Hospital, London, GBR; 2 Trauma and Orthopaedic Surgery, Princess Royal University Hospital, King's College Hospital NHS Foundation Trust, London, GBR; 3 Cardiology, Frimley Park Hospital, Frimley Health NHS Foundation Trust, Frimley, GBR; 4 Urology, Wrexham Maelor Hospital, Wrexham, GBR; 5 Emergency Medicine, Sharq Elmadina Hospital, Egyptian Ministry of Health, Alexandria, EGY; 6 Internal Medicine, Frimley Park Hospital, Frimley Health NHS Foundation Trust, Frimley, GBR; 7 Orthopaedic Surgery, Frimley Health NHS Foundation Trust, Camberley, GBR; 8 Paediatric Surgery, Mansoura University Children Hospital, Mansoura, EGY; 9 General Medicine, Princess Royal University Hospital, King's College Hospital NHS Foundation Trust, London, GBR; 10 Orthopaedics, North Bristol NHS Foundation Trust, Bristol, GBR

**Keywords:** artificial intelligence (ai), imaging analysis, implant selection, joint replacement surgery, orthopedic, real-time adaptation, robotic-assisted surgery

## Abstract

Artificial intelligence (AI) is affecting the practice of orthopedic surgery, offering innovative solutions from preoperative planning to intraoperative guidance and real-time adaptation. This review highlights current advancements in AI-driven imaging analysis, anatomical segmentation, and implant selection, highlighting improvements in surgical precision, efficiency, and patient-specific customization. Intraoperatively, AI enables real-time image processing, integration with robotic systems, and adaptive feedback mechanisms that enhance accuracy, reduce complications, and personalize care. Clinical applications span joint replacement, spine, and trauma surgery, where AI supports diagnosis and offers support in decision-making, often surpassing conventional methods. Despite these promising developments, challenges remain regarding data quality, model generalizability, transparency, and ethical considerations. Future directions emphasize explainable AI, multimodal data integration, and closer synergy between AI, robotics, and digital health to advance personalized orthopedic care.

## Introduction and background

Artificial intelligence (AI) is transforming surgical decision-making in orthopedics by enhancing the accuracy of diagnosis and making precise decisions. Recent reviews and studies highlight the integration of AI across the perioperative continuum, from preoperative imaging analysis and risk prediction to intraoperative guidance and postoperative monitoring [1-4].

Machine learning (ML) and deep learning models, including advanced architectures such as convolutional neural networks (CNNs) and ensemble learning methods, have shown and proved significant advantages across a wide range of medical imaging and diagnostic tasks. These applications encompass, but are not limited to, the accurate detection of fractures and precise identification and classification of orthopedic implants, as well as the prediction of clinical outcomes based on patient-specific data and imaging findings [5-9]. AI-driven decision aids have also been shown to improve shared decision-making and patient satisfaction in clinical trials [10].

Despite advancements in orthopedic surgery using AI, there remains a need for external validation, model interpretability, data standardization, and consideration of ethical issues such as bias and accountability [2,11-13]. In this review, we comprehensively highlight the literature findings regarding the various applications of AI in orthopedic practice.

## Review

Methodology

This work was conducted as a comprehensive review aiming to synthesize the current evidence regarding the role of AI in orthopedic surgical decision-making, from preoperative planning to intraoperative decision support for surgeons and real-time adaptation. Relevant literature was identified through a comprehensive search of PubMed, Scopus, Web of Science, and Google Scholar databases up to June 2025. Search terms included combinations of keywords such as "artificial intelligence", "machine learning", "deep learning", "orthopedic surgery", "preoperative planning", "intraoperative guidance", "robotic-assisted surgery", and "real-time feedback".

The selection of studies was based on their relevance to the topic and contribution to understanding clinical applications, benefits, limitations, and future research regarding this topic. No restrictions were applied regarding study design, and both experimental and observational studies, as well as high-quality review articles, were included.

Reported systematic review protocols, such as PRISMA (Preferred Reporting Items for Systematic Reviews and Meta-Analyses), were not applied, and no quantitative meta-analysis was performed. Instead, the included literature was thematically organized into key domains to help evaluate the reporting literature: (1) AI in preoperative planning, including imaging analysis, anatomical segmentation, and implant selection; (2) intraoperative AI-guided systems; (3) real-time adaptive feedback; and (4) subspecialty-specific applications in joint replacement, spine, and trauma surgery. The synthesis emphasizes technological advancements, clinical outcomes, and existing challenges, providing an integrated perspective on AI's evolving role in orthopedic surgical practice.

Preoperative planning with AI in orthopedic surgeries

AI has changed and improved preoperative planning in orthopedic surgery through 3D reconstruction and virtual planning features for patients [14].

Imaging Analysis and Anatomical Segmentation

Modern AI algorithms utilize deep learning techniques to automatically generate highly accurate three-dimensional models from CT and MRI scans, helping surgeons to perform highly precise interventions through orthopedic operations. These AI-driven systems can reconstruct 3D bone models in significantly reduced timeframes, with reconstruction processes taking approximately 233 seconds compared to traditional manual methods that require 30 minutes or more. The integration of AI algorithms has addressed critical limitations of conventional computer vision methods, including time-intensive processes, artifact-induced quality degradation, and limited accuracy in non-contrast imaging [14,15].

CT- and MRI-based modeling provide detailed anatomical information essential for making optimum decisions in orthopedic surgery. AI algorithms excel at processing vast amounts of imaging data from CT scans and MRI sequences, automatically segmenting anatomical structures with remarkable precision. The AI KNEE software (version 3.0, Longwood Valley Technology, Beijing, China), for example, shows how to measure important parameters like mechanical axes, anatomical axes, and rotational angles while automatically segmenting bones and producing 3D models of the entire lower limb, femur, and tibia. These advanced modeling techniques enable surgeons to assess patient-specific anatomy comprehensively, identifying anatomical variations and pathological conditions that influence surgical planning and implant selection [16].

Deep learning, particularly CNNs, has greatly advanced anatomical landmark detection, enabling automatic and precise localization comparable to human experts. Studies show high accuracy in detecting multiple spinal landmarks and strong agreement with manual measurements. Ensemble models, such as Cascaded Pyramid Networks with DSNT (Differentiable Spatial to Numerical Transform) layers, further enhance coordinate regression and maintain robust performance across various pathologies [17,18].

AI-driven automated segmentation, particularly with U-Net-based deep learning models, performs better than manual techniques, attaining over 98% accuracy, high Dice coefficients (0.986), and low surface errors (0.234 mm), even in complex fractures. These techniques are widely used in biomedical imaging because they minimize variability, save time, and guarantee consistent, repeatable outcomes for trustworthy preoperative planning [19,20].

Implant Selection and Sizing

AI-assisted implant selection algorithms represent a paradigm shift in orthopedic implant selection, utilizing ML models to optimize implant choice based on patient-specific characteristics. These sophisticated algorithms analyze extensive datasets comprising patient demographics, anatomical measurements, and historical surgical outcomes to predict optimal implant specifications. Research demonstrates that AI models can achieve femoral and tibial implant size prediction accuracy rates of 82.2% and 85.0%, respectively, compared to conventional manufacturer default plans showing 68.4% and 73.1% accuracy. The integration of multiple discrete, ordinal, and continuous variables into predictive models enables real-time refinement and accommodation of changing surgical, implant, and patient factors [21,22].

Patient-specific instrumentation (PSI) optimization through AI technologies has emerged as a powerful tool for enhancing surgical precision and efficiency [23]. AI algorithms analyze patient-specific anatomical data to design customized surgical guides and instrumentation that perfectly match individual anatomical variations. ML models demonstrate the capability to reduce surgeon corrections to preoperative plans by 39.71% compared to manufacturer default plans, with improvements reaching 47.95% for surgeons who frequently modify standard templates. The AI-driven PSI approach enables the creation of surgeon-specific and patient-specific preoperative plans that incorporate individual surgeon preferences learned from previous cases, thereby reducing planning time and improving surgical workflow efficiency [21].

Comprehensive comparative studies consistently demonstrate the superior performance of AI-based planning over traditional templating methods in orthopedic surgery. A prospective study comparing AI 3D planning with traditional 2D template measurements revealed significantly higher accuracy rates, with AI achieving 91.67% accuracy for femoral components compared to 66.67% for traditional methods. Similarly, tibial component accuracy reached 87.50% with AI versus 62.50% with conventional templating. These improvements translate into measurable clinical benefits, including reduced operation time, decreased intraoperative blood loss, lower postoperative drainage volumes, and improved patient-reported outcomes. With complete coincidence rates of 90% for femoral prostheses and 86.7% for tibial prostheses, the AI-based method also performs better in predicting prosthesis size and axial alignment [16,24].

**Figure 1:**
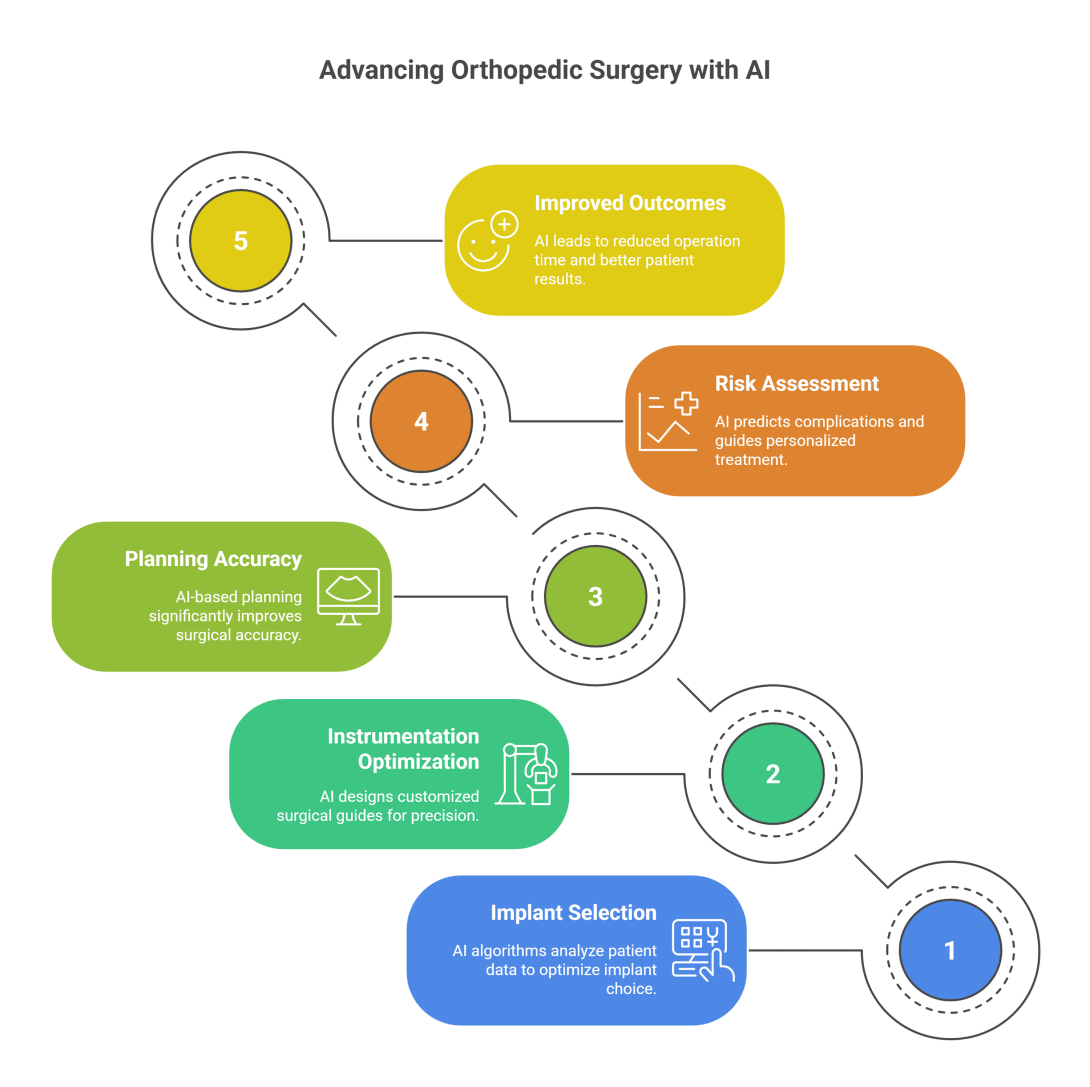
Preoperative planning in orthopedic surgery using artificial intelligence Figure credit: Momen Abdelglil Source: [14-24]

The use of AI-based systems preoperatively is not only specific for implant selection but also integrated in the risk assessment. ML models analyze vast datasets of historical surgical cases to identify patterns and correlations that inform optimal surgical strategies. These predictive systems can forecast potential complications, estimate implant longevity, and guide personalized treatment approaches based on patient-specific risk factors. Surgeons can make evidence-based decisions that maximize both short-term surgical results and long-term implant survival by incorporating predictive analytics into preoperative planning workflows. This ultimately improves patient satisfaction and lowers revision rates. Through ongoing learning from new cases, sophisticated AI algorithms keep improving over time, increasing their clinical utility and predictive power (Figure 1) [14,21,22,24].

Intraoperative guidance systems

Edge AI allows for on-device, real-time image segmentation during robotic-assisted orthopedic surgeries, minimizing latency and reliance on external servers. Deep learning architectures such as U-Net, DeepLabV3, and transformer-based models, when optimized for edge deployment, can achieve sub-100 ms inference times while maintaining high segmentation accuracy. This supports rapid, accurate anatomical identification and tool guidance directly at the surgical site, enhancing surgical precision and patient outcomes [25].

Real-Time Image Processing and Analysis

Real-time ultrasound imaging, combined with CNNs and GANs, enables robust, computationally efficient segmentation of bone surfaces and bone shadows. These methods achieve high accuracy (F-scores above 95% and localization errors as low as 0.11-0.2 mm), making ultrasound a viable, radiation-free alternative for intraoperative guidance in computer-assisted orthopedic surgery [26,27].

Mixed reality (MR) and augmented reality (AR) systems overlay real-time processed images and 3D models onto the surgical field, providing intuitive, interactive guidance. These technologies improve spatial awareness, communication, and surgical accuracy and are increasingly integrated with AI-driven image analysis for enhanced intraoperative decision-making [28-30].

AI-Driven Robotic-Assisted Surgery in Orthopedics

AI-powered robotic systems, such as Stryker's Mako and TiRobot, leverage real-time imaging and preoperative models to achieve sub-millimeter accuracy in implant positioning and bone cuts. This results in improved alignment, reduced soft-tissue damage, and fewer surgical complications. Clinical studies report a reduction of up to 30% in operative time, 35% less blood loss, and faster patient recovery compared to conventional methods [31,32]. AI-driven feedback also allows for dynamic adjustments during surgery, further improving reproducibility and patient outcomes [31-33].

**Figure 2:**
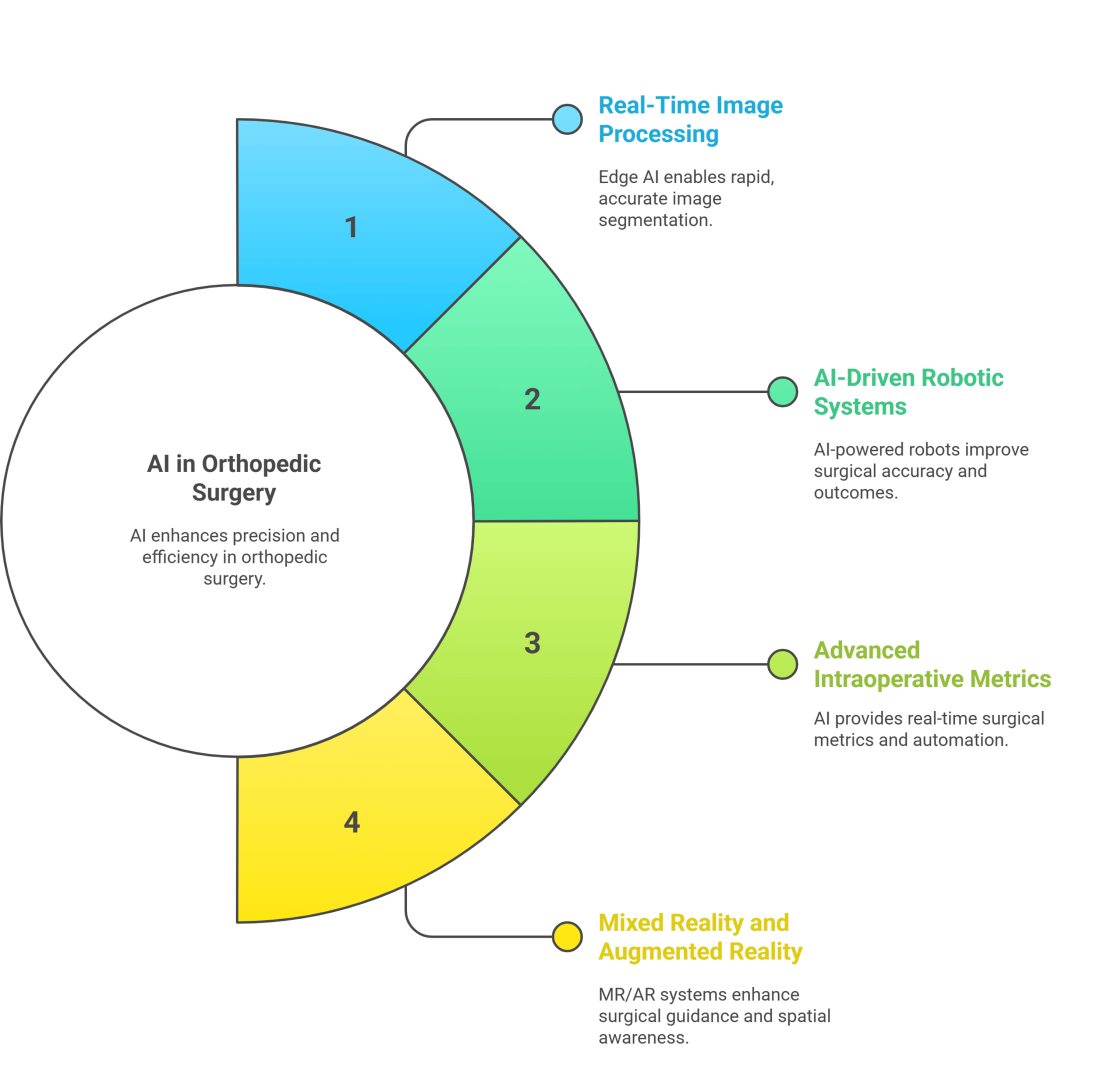
The use of AI in intraoperative guidance Figure credit: Momen Abdelglil Source: [25-36]

AI enables advanced intraoperative metrics, including force and tactile measurements, and can automate certain surgical steps. ML algorithms process sensor and imaging data to guide instrument placement, detect anatomical structures, and even predict surgical outcomes. AR and haptic feedback systems further enhance the surgeon's situational awareness and control (Figure 2) [32,34-36].

Real-time adaptation and feedback systems

Modern systems integrate advanced sensors, AI, and ML to deliver real-time feedback during procedures. For example, sports tracking technologies, such as thermal imaging, trajectory tracking, and acoustic feedback, have been adapted into unified systems to improve bone alignment, implant placement, and fracture fixation, thereby reducing iatrogenic errors and improving patient outcomes [37]. AI-driven platforms analyze intraoperative data, predict complications, and adapt protocols instantly, shifting orthopedic care from retrospective analysis to dynamic, individualized decision-making [38,39].

Deep learning-based acoustic sensing can detect critical events like drill breakthrough in milliseconds, preventing injury to vital structures and outperforming human reaction times [37]. Smart implants and load-sensing devices provide continuous, objective feedback on structural integrity and biomechanics postoperatively, enabling tailored rehabilitation and early complication detection [1,40]. Virtual reality (VR) and robotic systems with real-time visual, haptic, and auditory feedback accelerate skill acquisition, improve technical accuracy, and reduce errors, especially for novice surgeons [36,41-43].

These systems have demonstrated significant benefits, including reduced error rates, improved technical skills, and more personalized patient care [36,37,39,41]. The integration of MR and AR further enhances intraoperative visualization and communication, supporting more accurate and efficient procedures [29,30].

Clinical applications of AI by subspecialty

AI tools can significantly identify implants, detect failures, and measure dimensions from radiographs, thereby helping in the preoperative planning process and revisions while reducing errors. However, most lack external validation and explainability, underscoring the need for standardized development and reporting [44].

Joint Replacement Surgery

AI-driven systems can analyze clinical data to generate personalized risk and benefit predictions for joint replacement. This approach aims to reduce inappropriate surgeries and improve satisfaction by tailoring interventions to individual needs [45].

The convergence of AI with advanced imaging and robotic technologies is enhancing the precision, safety, and efficiency of joint replacement procedures. AI assists in optimizing implant positioning and surgical workflows, potentially lowering costs and accelerating the adoption of innovative techniques. Ongoing collaboration among stakeholders is essential to fully realize these benefits [46,47]. AI, combined with 3D printing, enables the design and fabrication of patient-specific prosthetic joints, improving biomechanical function and reducing surgical risks compared to standard devices [48].

Major Applications of AI in Spine Surgery

AI-driven tools excel at interpreting spinal imaging, reliably identifying fractures, stenosis, and tumors, and even reconstructing 3D spine images while reducing radiation exposure. These technologies support earlier and more accurate diagnoses, especially in complex spinal anatomy [49-52].

AI is integrated with robotic systems to assist in pedicle screw placement, achieving high accuracy (up to 97%) and improving safety, although this sometimes comes at the cost of increased operative time. AI also aids in preoperative planning, navigation, and real-time intraoperative decision-making, often in combination with augmented or virtual reality [52-56].

Applications of AI in Trauma Surgery

AI models can predict the need for major surgery using prehospital data (vital signs, clinical scores) with high accuracy, enabling better triage and resource allocation before hospital arrival [57]. AI and ML are used to predict outcomes, assess injury severity, forecast transfusion needs, detect hemorrhage, and predict coagulopathy. These models often outperform traditional methods in retrospective studies, but most lack external validation and are not yet widely used in clinical practice [58]. It is also used to analyze large datasets to optimize trauma center locations, predict patient flow, and adapt staffing models, improving access and quality of care across trauma networks. However, broader implementation is limited by cost, expertise, and stakeholder buy-in [59].

**Figure 3:**
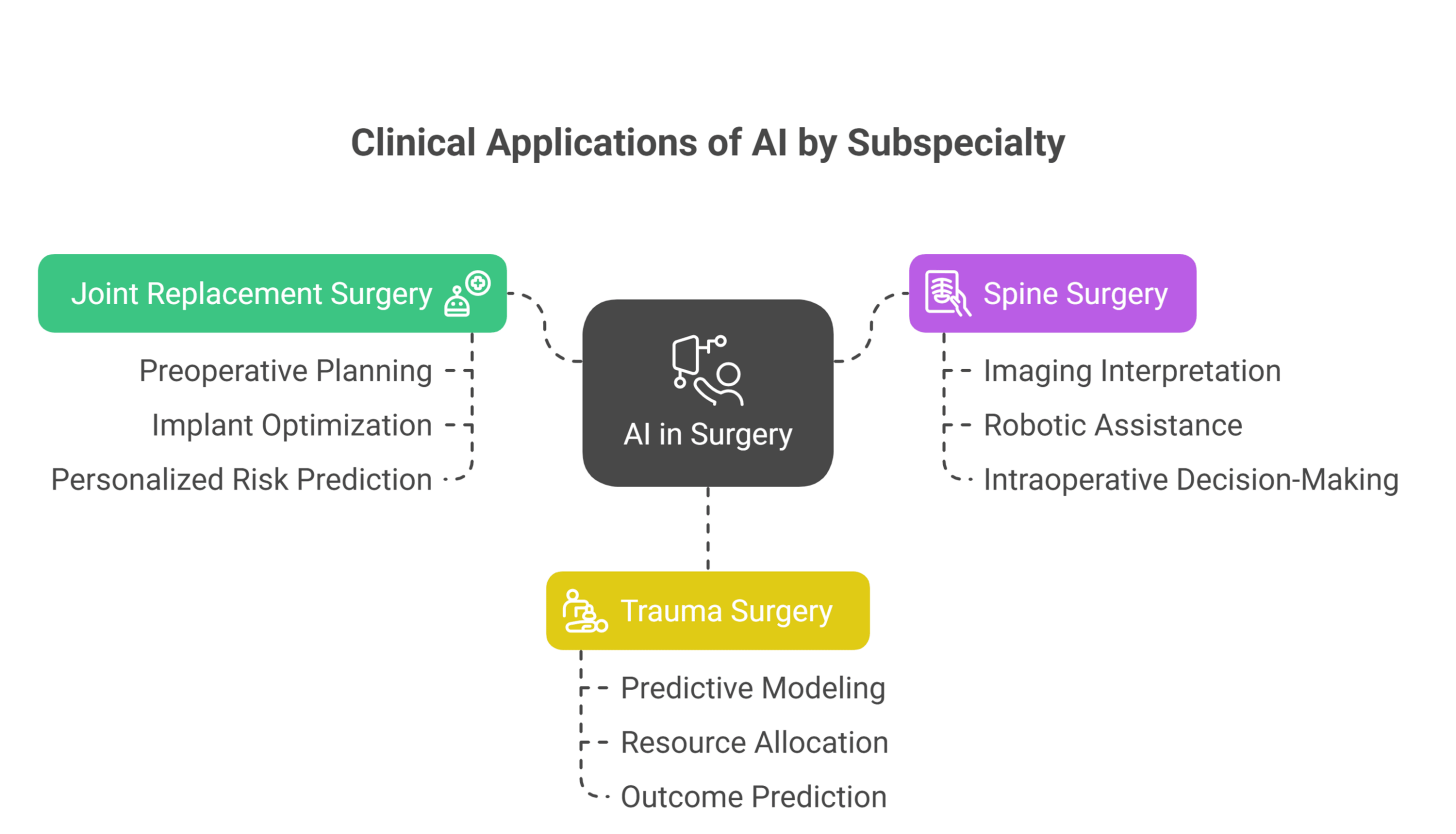
Clinical applications of AI by subspecialty Figure credit: Momen Abdelglil Source: [44-60]

Surgeons recognize AI's potential to support decision-making in trauma and emergency contexts, especially for handling complex, high-volume data. However, concerns remain about model transparency, bias, integration into workflows, and the need for rigorous validation before routine use (Figure 3) [60].

Current limitations and future directions

Despite these advances in the use of AI in current practice, there are several limitations, including data quality issues, lack of external validation, limited generalizability, and ethical concerns such as bias and transparency [2,61-63]. Current research highlights the need for significant validation frameworks, standardized reporting, and interdisciplinary collaboration to ensure safe and effective clinical integration [1,2,62,64].

Clinical adoption is significantly hindered by ethical concerns, including algorithmic bias, data privacy, and a lack of transparency [1,61]. Clear guidelines for the validation and application of AI tools in clinical practice are necessary, as regulatory frameworks continue to change [32,65].

**Figure 4:**
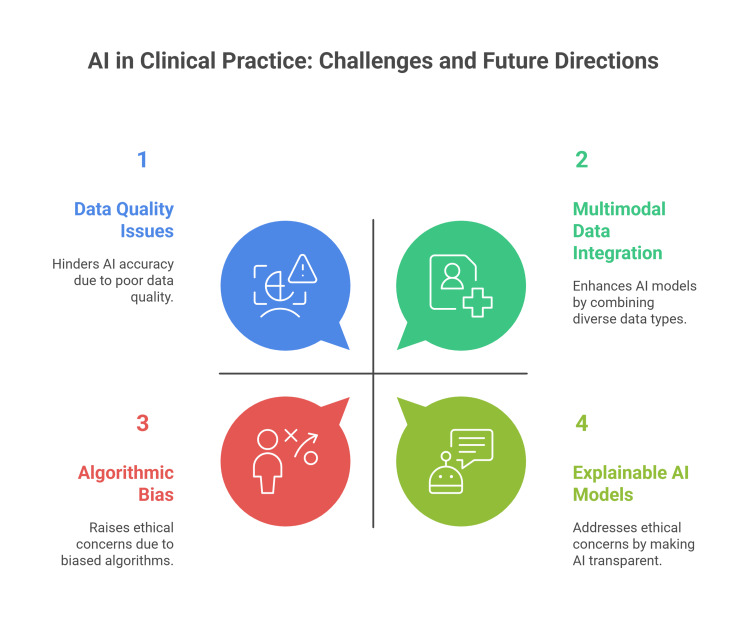
Current limitations and future directions for the use of AI in orthopedic surgery Figure credit: Momen Abdelglil Source: [1,2,7,62,64,65]

Future research is focusing on improving data quality through multicenter studies, developing explainable and transparent AI models, integrating multimodal data (including imaging, clinical, and genomic data), and combining AI with robotics systems for personalized care (Figure 4) [4,7,32].

## Conclusions

AI is changing the practice of orthopedic surgery, enhancing the precision of diagnosis and prognosis, optimizing preoperative planning, and enabling precise intraoperative execution through integration with advanced imaging, robotic platforms, and real-time feedback systems. These innovations have improved surgical precision, reduced complication rates, personalized treatment strategies, and enhanced patient outcomes reported by various specialties.

Furthermore, several limitations are observed, especially in data quality, external validation, model interpretability, and ethical considerations related to bias and privacy. To guarantee safe, equitable, and successful adoption, these issues must be resolved through standardized development frameworks, strong multicenter partnerships, and clear regulations. Orthopedic care could become a more outcome-driven, personalized, and predictive field as AI technologies advance and combine with robotics, digital twins, and multimodal health data.
